# Why physicians underuse patient-reported outcomes in atopic dermatitis and chronic urticaria — Insights from the UCARE/ADCARE PROMUSE study^☆^

**DOI:** 10.1016/j.waojou.2026.101398

**Published:** 2026-06-05

**Authors:** Ivan Cherrez-Ojeda, Karla Robles-Velasco, Ana Giménez-Arnau, Kiran Godse, Dorota Krasowska, Joanna Bartosińska, Paulina Szczepanik-Kułak, Bartłomiej Wawrzycki, Pavel Kolkhir, Anastasiia Allenova, Andrey Allenov, Sergey Tkachenko, Natasa Teovska Mitrevska, Dragan Mijakoski, Sasho Stoleski, Marta Kolacinska-Flont, Izabela Kuprys-Lipinska, Joanna Molinska, Alicja Kasperska-Zając, Magdalena Zajac, Mateusz Zamlynski, Florin Mihaltan, Ruxandra Ulmeanu, Anna Zalewska-Janowska, Katarzyna Tomaszewska, Mona Al-Ahmad, Maryam Ali Al-Nesf, MSs ABHS, Tayseer Ibrahim, Sami Agel, David Pesqué, Mónica Rodríguez-González, Guillermo Hideo Wakida-Kuzunoki, German D. Ramon, Gonzalo N. Ramon, Sophia Neisinger, Hanna Bonnekoh, Maia Rukhadze, Maryam Khoshkhui, Mehraneh Movahedi Aliabadi, Daria Fomina, Désirée Larenas-Linnemann, Mitja Košnik, Rabia Oztas Kara, Chrystopherson Gengyny Caballero López, Qiang Liu, Juan Carlos Ivancevich, Luis Felipe Ensina, Nelson Rosario, Violeta Kvedariene, Moshe Ben-Shoshan, Roberta Fachini Jardim Criado, Andrea Bauer, Annia Cherrez, Herberto Chong-Neto, Maria Isabel Rojo-Guierrez, Michael Rudenko, José Ignacio Larco Sousa, Aleksandra Lesiak, Edgar Matos, Nelson Muñoz, Jaime Moreno, Ivan Tinoco, Marco Faytong-Haro, Jean Bousquet, Torsten Zuberbier

**Affiliations:** a Institute of Allergology, Charité – Universitätsmedizin Berlin, Corporate Member of Freie Universität Berlin and Humboldt-Universität zu Berlin, Berlin, Germany; b Fraunhofer Institute for Translational Medicine and Pharmacology ITMP, Immunology and Allergology, Berlin, Germany; c Universidad Espiritu Santo, Samborondon, Ecuador; d Respiralab Research Group, Guayaquil, Ecuador; e Department of Dermatology, Hospital del Mar, IMIM, Universität Pompeu Fabra, Barcelona, Spain; f Department of Dermatology, D Y. Patil University School of Medicine, Mumbai, India; g Department of Dermatology, Venereology and Pediatric Dermatology, Medical University of Lublin, Lublin, Poland; h Department of Cosmetology and Aesthetic Medicine Medical University of Lublin, Lublin, Poland; i Laboratory of Immune-mediated Skin diseases, Institute of Regenerative Medicine, Biomedical Science & Technology Park, I.M. Sechenov First Moscow State Medical University (Sechenov University), Moscow, Russian Federation; j Institute for Leadership and Health Management, I.M. Sechenov First Moscow State Medical University (Sechenov University), Moscow, Russian Federation; k State Budgetary Healthcare Institution of the City of Moscow "City Polyclinic No. 166 of the Department of Health of the City of Moscow", Moscow, Russian Federation; l Federal State Budgetary Scientific Institution “NA Semashko National Research Institute of Public Health”, Moscow, Russian Federation; m Russian Medical Academy of Continuous Professional Education of the Ministry of Health of the Russian Federation, Moscow, Russian Federation; n Remedika General Hospital Dermatology Department, Skopje, Republic of North Macedonia; o International Balkan University (IBU), Skopje, Republic of North Macedonia; p Institute of Occupational Health of RN Macedonia, WHO CC, GA2LEN CC, Allergy Center, Skopje, Republic of North Macedonia; q Faculty of Medicine, SS Cyril and Methodius, University in Skopje, Skopje, Republic of North Macedonia; r Department of Internal Medicine, Asthma and Allergy, Barlicki Memorial Hospital Medical University of Lodz, Poland; s European Centre for Diagnosis and Treatment of Urticaria/Angioedema (GA2LEN UCARE /ACARE Network) and Department of Clinical Allergology and Urticaria of Medical University of Silesia, Silesia, Poland; t ARIA (Allergic Rhinitis and Its Impact on Asthma), Montpellier, France; u National Institute of Pneumology, Bucharest, Romania; v Medical University of Lodz, Psychodermatology and Neuroimmunobiology of the Skin Department, Lodz, Poland; w Microbiology Department, Faculty of Medicine, Kuwait University, Safat, Kuwait; x Allergy and Immunology Division, Medicine Department, Hamad Medical Corporation, Doha, Qatar; y Department of Dermatology, Hospital del Mar, Barcelona, Spain; z Universitat Autònoma de Barcelona (UAB), Barcelona, Spain; aa Hospital Español de México, Ciudad de México, México City, México; ab Colegio Mexicano de Pediatras Especialistas en Inmunología Clínica y Alergia, México; ac Instituto de Alergia e Inmunología del Sur, GA2LEN UCARE/Adcare/ACARE Centre, Bahia Blanca, Buenos Aires, Argentina; ad Centre Allergy & Immunology, Tbilisi, Georgia; ae Allergy Research Centre, Mashhad University of Medical Science(MUMS), Mashhad, Iran; af Moscow Practical and Research Centre of Allergy and Immunology, Clinical City Hospital, Moscow, Russian Federation; ag Moscow Department of Clinical Immunology and Allergology, I.M.Sechenov First Moscow State Medical University, Russian Federation Astana Medical University, Moscow, Russian Federation; ah Hospital Médica Sur, México City, México; ai Allergy University Clinic of Respiratory and Allergic Diseases, Golnik, Slovenia; aj Medical Faculty, University of Ljubljana, Slovenia; ak Department of Dermatology, Sakarya University Faculty of Medicine, Sakarya, Turkey; al Universidad Autónoma de Puebla, Hospital Universitario de Puebla, Servicio de Alergia e Inmunología Clínica, Puebla, Puebla, México; am Benemérita Universidad Autónoma de Puebla, Puebla, México; an Second Hospital of Hebei Medical University, Shijiazhuang City, Hebei Province, China; ao Servicio de Alergia e Immunologia Clinica Santa Isabel Buenos Aires, Argentina; ap Division of Allergy, Clinical Immunology and Rheumatology, Department of Pediatrics, Federal University of São Paulo and CP Alpha Clinical Research Centre, São Paulo, Brazil; aq Urticaria Centre of Reference and Excellence (UCARE), Federal University of Parana, Rua General Carneiro, Curitiba, Brazil; ar Institute of Biomedical Sciences, Department of Pathology, Faculty of Medicine, Vilnius University, Institute of Clinical Medicine, Clinic of Chest Diseases, Immunology and Allergology, Vilnius, Lithuania; as Division of Pediatric Allergy, Immunology and Dermatology, Department of Pediatrics, McGill University Health Center, Montreal, Quebec, Canada; at Faculdade de Medicina do ABC (FMABC), Santo André (SP), Brazil; au Department of Dermatology, University Allergy Centre, University Hospital Carl Gustav Carus, Technical University, Dresden, Germany; av Department of Dermatology, Allergology and Venereology, Charité – Universitätsmedizin Berlin, corporate member of Freie Universität Berlin and Humboldt-Universität zu Berlin, Berlin, Germany; aw Department of Pediatrics, Hospital de Clínicas, Federal University of Parana (UFPR), Curitiba, Brazil; ax Hospital Juarez De México, México City, México; ay London Allergy and Immunology Centre, London, United Kingdom; az Clinica San Felipe, Lima, Peru; ba Department of Dermatology, Pediatric Dermatology and Dermatological Oncology, Medical University of Lodz, Poland; bb Instituto Nacional de Salud del Nino, Lima, Peru; bc Specialist Centre: Muñoz Alergias y Pediatría, Riobamba, Ecuador; bd Centro Particular de Alergias, Ecuador; be Centro de Alergia Tinoco, Machala, Ecuador; bf Facultad de Investigación, Universidad Estatal de Milagro, Milagro, Guayas, Ecuador

**Keywords:** Atopic dermatitis, Barriers, Chronic urticaria, Dermatology, Patient-reported outcome measures

## Abstract

**Background:**

The recently published PROMUSE study performed by the GA^2^LEN UCARE (Urticaria Centers of Reference and Excellence) and ADCARE (Angioedema Centers of Reference and Excellence) networks found that patient-reported outcome measures (PROMs) are underused in clinical practice for atopic dermatitis (AD) and chronic urticaria (CU). The reasons for this remain unknown. We studied why physicians have reservations about using PROMs, how PROM use is affected by the barriers they perceive, and what the physician demographics/characteristics associated with these barriers are.

**Methods:**

This is an observational, cross-sectional study where the PROMUSE questionnaire was used and applied to physicians from 45 specialized centers worldwide. Of the 2534 physicians surveyed in the PROMUSE study, 474 who treated patients with AD and CU were included in the analysis.

**Results:**

For the 474 physicians who treat AD and CU patients, the most common issues perceived as primary barriers to PROM use were “time constraints” (n = 455/474), “not mandated to complete” (n = 428/474), and “patients dislike PROMs” (n = 425/474). Higher rates of barrier perception were observed for males, younger physicians, and non-specialist physicians. The more barriers physicians perceive, the less frequently PROMs are used, with a strong and significant negative correlation between the number of barriers perceived and the frequency of PROM use. Two of the 15 perceived barriers significantly prevented using PROMs: the belief that PROMs constrain the doctor-patient relationship and the belief that patients dislike them.

**Conclusions:**

The underutilization of PROMs in AD and CU is strongly driven by physicians' perceptions that patients dislike them and that they constrain the doctor-patient relationship. This suggests physicians may have deeper insights into the practical shortcomings of current instruments. Therefore, rather than solely focusing on physician education, existing AD and CU PROMs urgently require re-evaluation and co-development with direct patient involvement (eg, Core Outcome Sets) to ensure they are genuinely patient-centered and clinically relevant.

## Introduction

Chronic urticaria (CU) and atopic dermatitis (AD) cause substantial quality-of-life impairment, accounting for 4 and 12 million disability-adjusted life years (DALY) in 2021, which is 0.14% and 0.4% of the global health burden, respectively.^1^, ^2^, ^3^ Though not fatal, the chronicity and prevalence of these disorders make them major contributors within dermatology, underscoring the profound impact of symptoms such as itch, sleep disturbance, and psychological distress.^1^ Patient-reported outcome measures (PROMs) capture these impacts but remain rarely used. PROMs for allergic diseases need to be utilized more.^4^ Only 14% of physicians use PROMs for AD or CU, and half do so only “rarely” or “sometimes,” with the main barriers being time constraints (80–83%), patient dislike (52–60%), and lack of system integration (58–60%).

Our study seeks to elucidate how physician-related factors influence the implementation of PROMs in clinical practice by: 1) characterizing the physician profile, including individual attributes (eg, age, sex) and professional characteristics (eg, specialty, practice type, years of experience), associated to the perception of barriers; 2) assessing the extent to which these perceived barriers affect the frequency of PROM usage in CU and AD management; and 3) identifying whether certain barriers have a more significant impact than others.

## Methods

### Study design and settings

Information about the study design, participants, and the questionnaire has been described elsewhere.^4^ In brief, this was a global cross-sectional survey distributed across 72 medical centers in 73 countries.

### Study participants and network contributions

Participants were recruited from several established global networks specializing in allergic and inflammatory diseases:

Specialized Centers: 45 centers were affiliated with the Urticaria Centers of Reference and Excellence (UCARE), Atopic Dermatitis Centers of Reference and Excellence (ADCARE), and Angioedema Centers of Reference and Excellence (ACARE) networks.

General and Regional Networks: 28 centers consisted of physicians affiliated with the Allergic Rhinitis and Its Impact on Asthma (ARIA) network and various Latin American centers.

### Inclusion and exclusion criteria

To ensure the expertise of the respondents, inclusion was limited to physicians who actively treat AD and/or CU in their clinical practice. Specifically, for physicians recruited via the ARIA network, inclusion was restricted to allergists who confirmed they manage AD/CU during consultations. The survey was further disseminated through these centers to local and regional networks, encompassing dermatology and allergology clinics to capture a broad range of clinical settings.

### Statistical power and sample size

This study is an exploratory secondary analysis of the PROMUSE dataset.^4^ The final sample size of N = 474 was determined by the number of physicians from the parent study who met the inclusion criteria for AD and CU. To ensure the stability of the multivariable regression models, sample adequacy was assessed using the Events Per Variable (EPV) criterion. Given the high prevalence of the primary barriers (event rates >85%, resulting in >400 “events”), the sample provided sufficient power to include our selected independent predictors while maintaining an EPV ratio significantly higher than the recommended 10:1 or 15:1 threshold for stable parameter estimation.

### Handling of data integrity and quality control

To ensure rigorous data integrity and prevent duplicate responses across the participating global networks (UCARE, ADCARE, ACARE, and ARIA), the survey was administered using the QuestionPro platform (QuestionPro Inc., Seattle, WA, USA). The "Anti-Ballot Box Stuffing" (ABBS) security feature was enabled to restrict multiple submissions from a single participant. This was implemented primarily through browser-based cookie tracking, which identifies if a survey has already been completed on a specific device. Additionally, redundant responses were monitored via IP address filtering and a manual review of participant demographics (eg, name, institution, and email). This multi-layered approach was chosen to ensure that while duplicate entries were blocked, multiple unique experts operating within the same shared institutional network or hospital facility could still provide individual, valid responses.

### Variables

The variables included in the present study were: 1) Frequency of use: Physicians were asked how often they use PROMs, with 5 answer options (never, rarely, sometimes, often, and always); 2) Barriers to using PROMs: For 15 barriers, physicians were asked, “What are the main barriers to using PROMs?” Response options were Yes or No; and 3) Demographic data. It is important to note that the questionnaire utilized for this study was not formally validated prior to deployment. Furthermore, the survey design relied on a binary format and did not include free-text response options, which precluded the collection of qualitative or nuanced explanations from physicians regarding their perceptions or the specific shortcomings of existing PROMs.

### Descriptive analysis

The perception of each barrier was analyzed using the chi-square test of independence for its association with physician characteristics (gender, age, practice type, specialty, and years of experience).

### Logistic regression

The most frequent barriers to the use of PROMs perceived by physicians were used as dependent variables of logistic regression models to analyze the impact of sex, age, type of consultation, specialty type (specialist, family medicine doctor, dermatologist, pulmonologist, or other), and years of being a specialist. Specifically, binary logistic regression was used to assess the likelihood of using PROMs (use vs. no use) linked to the number of barriers perceived (from 0 to 15), sex (male or female), age (20–29, 30–39, 40–49, 50–59, 60+ years), type of consultation (public, private, or both), specialty (specialist, family medicine, pediatrics, allergist, dermatologist), and years of experience as a specialist (9 or less, 10–19, 20–29, 30+ years).

### Ordinal logistic regression

Ordinal logistic regression was used to assess the relationship between the frequency of using PROMs (never, rarely, sometimes, often, always) and perceived barriers and physician characteristics, which are all categorical variables. This model provided odds ratios (ORs) indicating the likelihood of being in a higher frequency category for using PROMs for each level of the categorical predictor compared to the reference level, holding all other variables constant. The proportional odds assumption was tested and confirmed to be satisfied, supporting the validity of the ordinal logistic regression analysis in this context.

### Ethics review

This study adhered to the World Medical Association's Helsinki Declaration on Ethical Conduct and was approved by the Guayaquil, Ecuador IRB's “Comité de ética e Investigación in Seres Humanos (CEISH).” All participants in this study provided informed consent.

## Results

Similar to findings in our PROMUSE analysis, across 474 physicians who treat AD and CU patients, the perception of barriers was nearly universal. The 7 most prevalent issues were identified by 88.8%–95.9% of respondents. These included time constraints (n = 455/474; 95.9%), lack of mandate (n = 428/474; 90.2%), and patient-related factors, such as the perception that patients dislike PROMs (n = 425/474; 89.6%). Other significant structural barriers included a lack of clinical system integration (n = 425/474; 89.6%), unavailability in native languages or for specific age groups (n = 425/474; 89.6%), and the belief that disease is sufficiently understood without PROMs (n = 422/474; Supplemental Table 1). For more details of descriptive results for all barriers, see Supplemental Table 2.

### Physicians less likely to perceive barriers: female, older, those working in public/private systems

In multivariable logistic regression, several physician characteristics were independently associated with the perception of barriers to PROM use in CU and AD. Female physicians were less likely than males to perceive “not mandated to complete” (OR = 0.63, p < 0.05) and “uncertainty about reliability” (OR = 0.67, p < 0.1). Compared with the youngest group (20–29 years), older physicians reported markedly fewer barriers, particularly for time constraints (OR = 0.08–0.39, all p < 0.05). Physicians working in both public and private systems were less likely to perceive “patients dislike PROMs” (OR = 0.52, p < 0.01) but more likely to report age-group limitations (OR = 1.73, p < 0.05) and unsuitability for obtaining information (OR = 1.66, p < 0.05), see Supplemental Table 3.

### Allergists and dermatologists reported fewer perceived barriers

Specialty differences were prominent. Allergists consistently reported fewer barriers, including time constraints, mandates, additional costs, constraints of the doctor–patient relationship, lack of confidence, and tool complexity (all ORs <0.65, p < 0.05). Dermatologists also perceived fewer barriers in several domains, whereas family physicians and pediatricians were more likely to report “sufficient understanding without PROMs” and age-related unavailability. Specialist status itself was associated with increased odds of reporting time constraints (OR = 3.24, p < 0.05) but fewer concerns about interpretation (OR = 0.43, p < 0.05).

### The more barriers physicians perceive, the less often they use PROMs

**Fig. 1:**
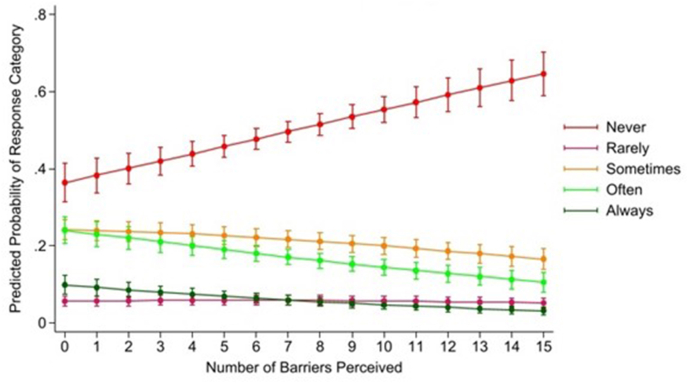
**The predicted probabilities of healthcare providers' frequencies of using PROMs are based on the number of perceived barriers.** PROMs = patient-reported outcome measures. The graph, derived from an ordinal logistic regression analysis, shows the predicted probabilities of healthcare providers' frequencies of using PROMs based on the number of perceived barriers. As the number of obstacles increases from 0 to 15, the probability of providers reporting “Never” using PROMs rises significantly, while the probabilities for “Rarely,” “Sometimes,” “Often,” and “Always” decrease. This inverse relationship highlights that more perceived barriers lead to less frequent use of PROMs, indicating the importance of addressing these barriers to enhance PROM utilization among providers

The number of barriers to the use of PROMs perceived by physicians was correlated to the frequency of their use. Across all physicians, 64% reported that they do not use PROMs for AD or CU. For physicians who do not have any of the 15 barriers, the rate of never using PROMs was 36.4%, significantly lower compared to 62.8% of physicians who see all 15 barriers as such (Fig. 1). With each additional perceived barrier to using PROMs, the rate of physicians who do not use them increased by 2.8% (Table 1).

**Table 1:** **Factors influencing PROMs use and frequency of use based on the number of barriers**.

VARIABLES	Binary logistic regression use of PROMs	Ordinal logistic regression PROM frequency of use
	OR (SE)	OR (SE)
Total barriers (0–15)	0.972^a^ (0.0167)	0.921^c^ (0.0136)
Sex (ref = male)		
Female	1.330^b^ (0.175)	1.099 (0.121)
Age group (Ref = 20–29)		
30-39	0.954 (0.221)	1.082 (0.216)
40-49	0.958 (0.276)	1.241 (0.303)
50-59	0.979 (0.344)	0.918 (0.271)
60+	1.172 (0.501)	0.868 (0.313)
Type of consultation (Ref = Public practice)		
Private practice	1.148 (0.204)	0.879 (0.132)
Both public and private	1.170 (0.174)	1.000 (0.122)
Specialist	0.569^c^ (0.119)	1.049 (0.185)
Family medicine	0.761 (0.202)	0.694^a^ (0.138)
Pediatrics	1.099 (0.196)	0.745^b^ (0.112)
Allergist	3.914^c^ (0.639)	2.400^c^ (0.315)
Dermatologist	6.501^c^ (1.165)	2.087^c^ (0.300)
Years as a specialist (Ref = 9 or less)		
10-19	0.879 (0.167)	0.930 (0.148)
20-29	0.650 (0.180)	0.802 (0.184)
30+	0.434^b^ (0.156)	1.113 (0.336)

OR = odds ratio; PROM = patient-reported outcome measure; Ref = reference; SE = standard error.

a p < 0.1.

b p < 0.05.

c p < 0.01

Ordinal logistic regression demonstrated a negative association between barrier count and frequency; each additional barrier was linked to a 7.9% lower likelihood of moving to a higher frequency category (eg, from “sometimes” to “often”) (OR = 0.921, p < 0.01), Fig. 1.

### Two perceived barriers significantly impede the use of PROMs

Two specific barriers were identified as primary drivers of underuse (Table 2). The likelihood of “always” using PROMs was significantly lower among physicians who believed “PROMs constrain the doctor-patient relationship” (OR = 0.734, 26.6% lower likelihood, p < 0.05) or that “patients dislike PROMs” (OR = 0.769, 23.1% lower likelihood, p < 0.05). Compared to those who do not, physicians who feel uncomfortable using PROMs were 22.5% less likely to use them always (OR: 0.775), but this was not statistically significant.

**Table 2:** **Ordinal logistic regression: the factors that affect the frequency of physicians' PROM use**.

VARIABLES	Ordinal logistic regression PROM frequency of use
**PROM-relatad barrier**	**OR (SE)**
Constraint doctor-patient relationship	0.734 (0.109)^a^
Patients dislike PROMs	0.769 (0.0948)^a^
Feel uncomfortable	0.775 (0.102)
Sufficient understanding without PROMS	0.872 (0.0993)
Lack of confidence in interpreting	0.872 (0.117)
Perceived as an additional cost	0.853 (0.120)
Too complicated to fill in	0.861 (0.123)
Lack of integration into clinical systems	0.862 (0.101)
Not available for specific age groups	0.937 (0.118)
Time constraints	0.905 (0.123)
Uncertainty about reliability	0.999 (0.124)
Not available in the native language	1.019 (0.127)
Mandated to complete	1.210 (0.145)
Too complicated to evaluate/score	1.081 (0.161)
Not suitable for obtaining the information	1.138 (0.169)
**Sex (Ref** = **Male)**	
Female	1.092 (0.122)
**Age group (Ref** = **20**–**29)**	
30-39	1.155 (0.235)
40-49	1.354 (0.336)
50-59	0.991 (0.296)
60+	0.929 (0.340)
**Type of consultation (Ref** = **public practice)**	
Private practice	0.849 (0.128)
Both public and private	0.959 (0.120)
Specialist	0.982 (0.178)
Allergist	2.418 (0.322)^b^
Dermatologist	2.070 (0.302)^b^
Pediatrics	0.723 (0.111)^a^
Family medicine	0.690 (0.140)
**Years as a specialist (Ref** = **9 or less)**	
10-19	0.923 (0.148)
20-29	0.806 (0.187)
30+	1.129 (0.344)

OR = odds ration; PROM = patient-reported outcome measure; Ref = reference; SE = standard error.

a p < 0.05.

b p < 0.01

## Discussion

The significance of patient-reported outcome measures (PROMs) in managing AD and CU is well recognized.^1^^,^^5^, ^6^, ^7^ However, PROM use rates are low, as demonstrated in our previous study, which found that PROMs are underused in clinical practice.^4^ Overall, we believe that physicians may need assistance in implementing greater use of PROMs.^4^ The present study analyzed a diverse population of physicians who treat patients with AD and CU, which confirmed and complemented our prior findings. Our results indicate that most physicians who treat CU and AD see many barriers to implementing PROMs, which negatively impact the frequency of use in physicians who use them.

Across the 7 most common issues perceived as primary barriers to using PROMs in CU and AD (ie, time constraints, lack of mandate, patients disliking PROMs, lack of integration into clinical systems, not being available in native languages or specific age groups, and sufficient understanding of disease without PROMs), 88%–95% of physicians see each as a problem. This was largely independent of physician demographics and other features, including work experience and settings. However, there was a trend toward higher rates of barrier perception in male vs. female physicians, younger vs. older physicians, and non-specialist vs. specialist physicians, especially allergists. This distinction may be explained by the differing roles of PROMs across clinical settings. While PROMs are an established and often mandated component of research, their perceived utility in clinical practice varies by discipline. In specialist services, requirements for auditing, benchmarking, and quality improvement often make PROM collection a necessity, which may account for the higher utilization rates observed among specialists in our study.

Physicians broadly view using PROMs for CU and AD as problematic for many reasons. Previous studies have demonstrated that physicians often view PROMs as a burden and see many problems with their use in clinical practice.^8^^,^^9^ Barriers to implementation included the perception that PROMs are intrusive to clinical practice, lack of infrastructure, fear that results may be used for cost containment and service eligibility instead of service quality improvement, difficulties with measures, ethical and confidentiality regulations, and web security and data management regulations.

In our study, family physicians and pediatricians were more likely to report "sufficient understanding without PROMs.” This reflects a broader reliance on clinical judgment over standardized tools, despite evidence of knowledge gaps in CU management. For instance, Cherrez et al^10^ found that only 19% of physicians were familiar with CU guidelines, while Kolkhir et al^11^ reported that even among those aware, 30% deviated from recommendations, and half of them relied on their own clinical experience. General practitioners themselves recognize deficiencies in their dermatology training across all career levels, which may further limit the adoption of guideline-based approaches.^12^ Similar patterns are seen in AD: Zucolo de Bortoli et al^13^ reported differences in management approaches depending on whether guidelines were followed, with dermatologists and allergists more often aligned on the use of newer therapies and systemic immunomodulators, while pediatricians tended to use corticosteroids and request food allergy investigations, and general practitioners demonstrate less expertise in AD, as reflected by fewer per capita visits and less frequent use of complex regimens.^14^ These discrepancies highlight how limited training and reliance on subjective understanding among family physicians and pediatricians can contribute to variability in managing chronic diseases such as AD and CU. PROMs provide essential insights about disease burden that cannot be captured by clinical impression alone; thus, greater efforts are needed to encourage their routine use among family physicians and pediatricians to align care with guideline-based, patient-centered practice.

Individual barriers were linked to distinct patterns of physician features, but these differences did not appear suitable to guide barrier-specific education among distinct physician populations. Importantly, physician education on PROMs for CU and AD must address the issues that physicians perceive as barriers and highlight the benefits of using PROMs. For example, while 95% of physicians see time constraints as a problem when implementing PROMs, the integration of PROMs has the potential to save time and streamline consultations to have more time for communicating with patients. Calkins et al^15^ have shown that without PROMs, physicians underestimated or failed to recognize 66% of disabilities reported by their patients and overstated functional impairment in 21% in which patients reported no disability. However, similar studies in dermatology and immunology are needed.

Where PROMs are perceived as challenging to implement, they are less used. This was expected, since physicians who see problems changing their clinical practice (ie, starting to use PROMs or using them more often) are hesitant to do so. Our results indicate that each additional perceived barrier was associated with a 3% lower likelihood of being a PROM user; conversely, a lower burden of perceived barriers may correlate with higher adoption rates. Notably, our research revealed that 36.4% of physicians who indicated no barriers yet reported never using PROMs. This indicates that the simple lack of perceived barriers is insufficient to guarantee clinical adoption. The “participation gap” may be attributed to unquantified structural factors not included in our 15-item list, such as insufficient institutional “nudges” or a lack of essential hardware (eg, tablets or integrated EHR modules) that a physician might not identify as a “barrier,” yet is crucial for workflow. Moreover, this may indicate “clinical inertia,” wherein clinicians adhere to conventional evaluation practices due to a lack of perceived therapeutic benefits that surpass their established routines. This disparity underscores that eliminating obstacles is merely half the challenge; fostering a favorable “pro-use” environment is equally essential.

Similarly, more perceived barriers were linked to an 8% lower frequency of use among those who do utilize them. However, given the cross-sectional nature of this study, we cannot infer directionality; it remains unclear whether the perception of barriers leads to lower use or if a lack of experience with PROMs contributes to a higher perception of barriers.

Most importantly, we identified 2 significant drivers of the underuse of PROMs in CU and AD: the perception that PROMs constrain the doctor-patient relationship and the belief that patients dislike them. It is crucial to ascertain whether these perceptions arise from insufficient physician education and familiarity with PROM implementation or the intrinsic limitations of the instruments themselves. Numerous studies have indicated that patients appreciate and like using PROMs and see them as an added value to their communication and relationship with their physicians. For example, the routine collection of PROMs in oncology has positively influenced patient-provider communication, significantly improving patient satisfaction. Furthermore, Velikova et al^16^ demonstrated that patients view PROMs as helpful in enhancing healthcare by promoting communication and fostering interpersonal connections. Chen et al^17^ demonstrated that PROM use had a significant beneficial effect on identifying unrecognized issues, and this practice enabled a more accurate diagnosis of diseases or patient conditions that may not be apparent, particularly in mental health, such as depression.^18^ In addition, Rubenstein et al^19^ highlighted the importance of integrating PROMs into clinical systems, resulting in improved emotional well-being scores, reduced reported social activity limitations among patients, and an increased rate of stress/anxiety diagnosis. In the other hand, taken together, these findings suggest that the perceived “patient dislike” reported by physicians may not reflect patients' actual experiences. Instead, this perception may stem from limited exposure to structured PROM implementation strategies or from negative experiences with poorly integrated tools. At the same time, not all PROMs are inherently patient-centered.^20^^,^^21^ Patients may perceive existing instruments as burdensome, overly lengthy, or surveys that appear detached from the patient's immediate clinical issues or that fail to capture outcomes that patients themselves consider most meaningful.^22^ In this context, the answer may encompass not just improved medical education but also the creation of more efficient, pertinent, and patient-centered tools that align with genuine patient objectives.

In CU and AD, there is currently a lack of robust evidence examining whether patient dissatisfaction with PROMs truly exists or whether it is predominantly a physician-derived perception. This represents an important gap in the literature and highlights the need for dedicated research in these populations.

The broader movement toward patient-centered measurement has accelerated the global shift from generic PROMs to Core Outcome Sets (COS). The Core Outcome Measures in Effectiveness Trials (COMET) initiative defines a COS as a consensus-based, standardized set of outcomes that should be measured and reported in all clinical trials within a specific health domain.^23^^,^^24^ Patients, caregivers, researchers, and healthcare professionals collaboratively establish COS, unlike traditional instrument development processes, ensuring that selected outcomes reflect shared priorities.^25^

In dermatology and allergy, the Cochrane Skin Group Core Outcome Set Initiative (CS–COUSIN) has advanced this paradigm by emphasizing that meaningful outcome measurement requires direct and sustained patient engagement.^26^ The success of this approach is evident in recent allergy-specific COS developments. For instance, the “COMFA” international Delphi consensus study for IgE-mediated food allergy involved over 800 participants, more than half of whom were patients and carers.^27^ Such initiatives underscore a critical transition: from clinician-driven metrics toward co-produced, patient-informed frameworks that better align research, clinical practice, and patient experience. Integrating such patient-driven sets into clinical practice for CU and AD could likely mitigate the “dislike” barrier identified in our results.

Finally, our finding that many physicians report discomfort in using PROMs further reinforces that underuse is likely multifactorial. Addressing these challenges necessitates not just improved tools but also systematic implementation techniques and teaching programs customized for clinical practice. These findings indicate that addressing the underutilization of PROMs in CU and AD may be supported by a dual approach: enhancing outcome measures for authentic patient relevance and providing clinicians with the requisite tools and confidence for effective integration into standard care.

Our study has several strengths and limitations. As for the former, we surveyed a sizeable and diverse population of physicians and used a comprehensive questionnaire. However, several limitations must be acknowledged. Most importantly, our sampling strategy primarily targeted physicians from specialized centers and academic settings. This focus may limit the representativeness of our findings and their generalizability to broader clinical contexts, particularly primary care, or non-academic community practices. Physicians in specialized or academic environments may employ PROMs more frequently due to institutional mandates, research requirements, or specialized infrastructure that is not available in general practice. In addition, the questionnaire utilized a binary yes/no format to identify barriers. While this provided a clear quantitative overview, a limitation of this approach is the lack of free-text responses, which might have allowed physicians to provide more nuanced explanations regarding the perceived validity of specific PROMs. Furthermore, our questionnaire was not validated; it did not include questions about PROM use according to disease severity or differentiation between PROMs for CSU and CIndU. Importantly, we should interpret these results cautiously because the cross-sectional nature of our study limits the ability to establish proper cause-and-effect relationships. Future studies should focus on the perception of PROMs by patients and whether apps that include PROMs, such as CRUSE, can help increase their use.^28^

In conclusion, while practical educational initiatives remain necessary, our findings indicate that physician reluctance is significantly rooted in the perceived negative impact of PROMs on patient experience. This highlights a critical need to re-evaluate the PROMs themselves, as physicians may possess valid insights into the shortcomings of current instruments in AD and CU. Addressing the underuse of PROMs in clinical practice requires a paradigm shift from forcing the adoption of potentially burdensome tools to actively involving patients in the co-creation and refinement of Core Outcome Sets (COS). Only by developing genuinely patient-centered instruments can we overcome the fundamental barriers of patient dissatisfaction and ensure clinical relevance.

## Disclosure of generative AI and AI-assisted technologies in the writing process

The authors declare that no generative artificial intelligence (AI) or AI-assisted tools were used in the writing, editing, data analysis, or preparation of this manuscript.

## Funding source

This study received general institutional funding from Charité-Universitätsmedizin Berlin (under no specific grant number), which has been instrumental in facilitating this research. The funder had no role in the study's design, execution, data analysis, interpretation, or preparation of the manuscript.

## Conflicts of interest

**Ana Giménez-Arnau** is or recently was a speaker and/or advisor for and/or has received research funding from Almirall, Amgen, AstraZeneca, Avene, Celldex, Escient Pharmaceuticals, Genentech, GSK, Instituto Carlos III- FEDER, Leo Pharma, Menarini, Novartis, Sanofi–Regeneron, Thermo Fisher Scientific, Uriach Pharma/Neucor; **Pavel Kolkhir** received Honoraria (advisory board, speaker)from Novartis, Roche and ValenzaBio, outside of submitted work; **Anastasiia Allenova** is a speaker for Novartis (outside of submitted work); **Marta Kolacinska-Flont** is a lecturer for Novartis; **Izabela Kuprys-Lipinska** is a lecturer for Novartis, Astrazeneca and GSK; **Hanna Bonnekoh** has received honoraria (advisory board, speaker) from AbbVie, Intercept Pharma, Novartis, Sanofi-Aventis and Valenza Bio Inc. outside the submitted work; **Juan Carlos Ivancevich** reports personal fees and non-financial support from Laboratorios Casasco, personal fees from Abbott Ecuador, personal fees from Laboratorios Bago Bolivia, personal fees from Faes Farma, outside the submitted work; **Luis Felipe Ensina** received financial support for lectures and clinical research from Novartis and Sanofi; **Violeta Kvedariene** reports non-financial support from Norameda, and Berlin CHemie Menarini, outside the submitted work; **Moshe Ben-Shoshan** is a consultant for Novartis and Sanofi; **Roberta Fachini Jardim Criado** has received Payment or honoraria for lectures, presentations, speaker's bureaus, manuscript writing or educational events: Takeda, Novartis, Sanofi, Pfizer, support for attending meetings and/or travel: Novartis, Sanofi, Abbvie, Pfizer, participation on a Data Safety Monitoring Board or Advisory Board: Pfizer, Lilly, Novartis, Abbvie; **Marcus Maurer** was a speaker, advisor, and/or received research funding from Allakos, Alvotech, Amgen, Aquestive, Aralez, AstraZeneca, Astria, Bayer, BioCryst, Blueprint, Celldex, Celltrion, Centogene, CSL Behring, Evoemmune, GSK, Ipsen, Kalvista, Kyowa Kirin, Leo Pharma, Lilly, Menarini, Mitsubishi Tanabe Pharma, Moxie, Noucor, Novartis, Orion Biotechnology, Pharvaris, Resonance Medicine, Sanofi/Regeneron, Septerna, Takeda, Teva, Trial Form Support International AB, Third HarmonicBio, Valenza Bio, Yuhan Corporation, and Zurabio. **The following authors have declared no conflicts of interest:** Drs Cherrez-Ojeda, Bousquet, Godse, Krasowska, Bartosińska, Szczepanik-Kułak, Wawrzycki, Allenov, Tkachenko, Teovska Mitrevska, Mijakoski, Stoleski, Sasho, Molinska, Kasperska-Zając, Zajac, Zamlynski, Mihaltan, Zalewska-Janowska, Tomaszewska, Al-Ahmad, Al-Nesf, Maryam, Ibrahim, Aqel, Pesqué, Rodríguez-González, Wakida-Kuzunoki, Both Drs Ramon, Neisinger, Rukhadze, Khoshkhui, Fomina, Larenas-Linnemann, Košnik, Mitja, Oztas Kara, Movahedi, Mehraneh, Caballero López, Liu, Rosario, Bauer, Chong-Neto, Rojo-Gutierrez, Rudenko, Larco, Sousa, Lesiak, Aleksandra, Matos, Edgar, Muñoz, Nelson, Tinoco, Ivan, Moreno, Jaime, Faytong-Haro, Robles-Velasco, Zuberbier.
