# Supplementary material for: Why physicians underuse patient-reported outcomes in atopic dermatitis and chronic urticaria — Insights from the UCARE/ADCARE PROMUSE study
Source: World Allergy Organ J. 2026 Jun 5;19(7):101398. doi: 10.1016/j.waojou.2026.101398 (PMC13264230; doi:10.1016/j.waojou.2026.101398)
Supplement: Multimedia component 1 [file mmc1.docx]

**Supplemental Table 1 (part 1/2). Physician populations that perceive the indicated barriers.**

| **The impact of physician gender, age, practice, specialty, and experience on the perception of barriers, n (%)** | | | | | | | | | |
| --- | --- | --- | --- | --- | --- | --- | --- | --- | --- |
| **Variables** | **All physicians** | **Time constraints** | **p-value** | **Not Mandated to complete** | **p-value** | **Patients dislike PROMs** | **p-value** | **Lack of integration into clinical systems** | **p-value** |
|  | (n=474) | (n=455) |  | (n=428) |  | (n=425) |  | (n=425) |  |
| **Sex** |  |  |  |  |  |  |  |  |  |
| **Male** | 170 (6%) | 162 (95%) |  | 168 (99%) |  | 152 (89%) |  | 164 (97%) |  |
| **Female** | 304 (64%) | 293 (96%) |  | 260 (86%) |  | 273 (90%) |  | 261 (86%) |  |
|  |  |  | 0.50 |  | 0.13 |  | **0.03** |  | 0.12 |
| **Age** |  |  |  |  |  |  |  |  |  |
| **20-29 years** | 54 (11%) | 54 (100%) |  | 54 (100%) |  | 54 (100%) |  | 54 (100%) |  |
| **30-39 years** | 170 (36%) | 168 (99%) |  | 162 (95%) |  | 167 (98%) |  | 152 (89%) |  |
| **40-49 years** | 116 (25%) | 109 (94%) |  | 104 (90%) |  | 100 (86%) |  | 103 (89%) |  |
| **50-59 years** | 80 (17%) | 73 (91%) |  | 59 (74%) |  | 59 (74%) |  | 75 (94%) |  |
| **60+ years** | 54 (11%) | 51 (94%) |  | 49 (91%) |  | 45 (83%) |  | 41 (76%) |  |
|  |  |  | 0.07 |  | **0.02** |  | **0.02** |  | 0.53 |
| **Types of consultation** |  |  |  |  |  |  |  |  |  |
| **Public** | 177 (37%) | 170 (96%) |  | 167 (94%) |  | 176 (99%) |  | 164 (93%) |  |
| **Private** | 98 (21%) | 96 (98%) |  | 86 (88%) |  | 89 (91%) |  | 80 (82%) |  |
| **Both** | 199 (42%) | 189 (95%) |  | 175 (88%) |  | 160 (80%) |  | 181 (91%) |  |
|  |  |  | 0.51 |  | 0.34 |  | **0.01** |  | **0.03** |
| **Specialty** |  |  |  |  |  |  |  |  |  |
| **Specialist** | 392 (83%) | 376 (96%) |  | 191 (49%) |  | 195 (50%) |  | 345 (88%) |  |
|  |  |  | 0.22 |  | **0.00** |  | **0.00** |  | 0.07 |
| **Family Medicine** | 28 (6%) | 25 (89%) |  | 20 (71%) |  | 24 (86%) |  | 27 (96%) |  |
|  |  |  | **0.01** |  | **0.02** |  | 0.71 |  | **0.01** |
| **Pediatrics** | 89 (19%) | 89 (100%) |  | 58 (65%) |  | 60 (67%) |  | 80 (90%) |  |
|  |  |  | 0.71 |  | 0.39 |  | 0.34 |  | 0.87 |
| **Allergist** | 187 (40%) | 168 (90%) |  | 58 (31%) |  | 54 (29%) |  | 164 (88%) |  |
|  |  |  | **0.01** |  | **0.00** |  | **0.02** |  | 0.14 |
| **Dermatologist** | 160 (34%) | 147 (92%) |  | 148 (93%) |  | 115 (72%) |  | 132 (83%) |  |
|  |  |  | 0.63 |  | 0.50 |  | **0.02** |  | 0.06 |
| **Years working as a physician** | |  |  |  |  |  |  |  |  |
| **1 to 9** | 191 (40%) | 191 (100%) |  | 183 (96%) |  | 164 (86%) |  | 178 (93%) |  |
| **10 to 19** | 141 (30%) | 132 (94%) |  | 103 (73%) |  | 121 (86%) |  | 124 (88%) |  |
| **20 to 29** | 74 (16%) | 68 (92%) |  | 74 (100%) |  | 73 (99%) |  | 61 (82%) |  |
| **30+** | 68 (14%) | 64 (94%) |  | 68 (100%) |  | 67 (99%) |  | 62 (91%) |  |
|  |  |  | 0.20 |  | **0.01** |  | **0.00** |  | 0.26 |

PROM=patient-reported outcomes measure

**Supplemental Table 1 (part 2/2). Physician populations that perceive the indicated barriers.**

| **The impact of physician gender, age, practice, specialty, and experience on the perception of barriers , n (%)** | | | | | | | |
| --- | --- | --- | --- | --- | --- | --- | --- |
| **Variables** | **All physicians** | **Not available in the native language** | **p-value** | **Not available for specific age groups** | **p-value** | **Sufficient understanding without PROMS** | **p-value** |
|  | (n=474) | (n=425) |  | (n=425) |  | (n=422) |  |
| **Sex** |  |  |  |  |  |  |  |
| **Male** | 170 (6%) | 159 (94%) |  | 157 (92%) |  | 160 (94%) |  |
| **Female** | 304 (64%) | 266 (88%) |  | 268 (88%) |  | 262 (86%) |  |
|  |  |  | 0.249 |  | 0.69 |  | 0.48 |
| **Age** |  |  |  |  |  |  |  |
| **20-29 years** | 54 (11%) | 51 (94%) |  | 50 (93%) |  | 53 (98%) |  |
| **30-39 years** | 170 (36%) | 158 (93%) |  | 157 (92%) |  | 162 (95%) |  |
| **40-49 years** | 116 (25%) | 109 (94%) |  | 103 (89%) |  | 91 (78%) |  |
| **50-59 years** | 80 (17%) | 61 (76%) |  | 72 (90%) |  | 65 (81%) |  |
| **60+ years** | 54 (11%) | 46 (85%) |  | 43 (80%) |  | 51 (94%) |  |
|  |  |  | **0.044** |  | 0.10 |  | **0.04** |
| **Types of consultation** |  |  |  |  |  |  |  |
| **Public** | 177 (37%) | 148 (84%) |  | 148 (84%) |  | 162 (92%) |  |
| **Private** | 98 (21%) | 94 (96%) |  | 79 (81%) |  | 76 (78%) |  |
| **Both** | 199 (42%) | 183 (92%) |  | 198 (99%) |  | 184 (93%) |  |
|  |  |  | 0.24 |  | **0.03** |  | 0.47 |
| **Specialty** |  |  |  |  |  |  |  |
| **Specialist** | 392 (83%) | 336 (86%) |  | 338 (86%) |  | 183 (47%) |  |
|  |  |  | **0.00** |  | **0.00** |  | **0.00** |
| **Family Medicine** | 28 (6%) | 23 (82%) |  | 27 (96%) |  | 26 (93%) |  |
|  |  |  | 0.19 |  | 0.97 |  | 0.52 |
| **Pediatrics** | 89 (19%) | 86 (97%) |  | 81 (91%) |  | 59 (66%) |  |
|  |  |  | 0.85 |  | **0.00** |  | **0.02** |
| **Allergist** | 187 (40%) | 132 (71%) |  | 157 (84%) |  | 61 (33%) |  |
|  |  |  | **0.00** |  | **0.00** |  | **0.01** |
| **Dermatologist** | 160 (34%) | 113 (71%) |  | 133 (83%) |  | 113 (71%) |  |
|  |  |  | **0.01** |  | 0.32 |  | 0.50 |
| **Years working as a physician** | |  |  |  |  |  |  |
| **1 to 9** | 191 (40%) | 183 (96%) |  | 182 (95%) |  | 176 (92%) |  |
| **10 to 19** | 141 (30%) | 133 (94%) |  | 112 (79%) |  | 106 (75%) |  |
| **20 to 29** | 74 (16%) | 59 (80%) |  | 68 (92%) |  | 73 (99%) |  |
| **30+** | 68 (14%) | 50 (74%) |  | 63 (93%) |  | 67 (99%) |  |
|  |  |  | **0.00** |  | **0.00** |  | 0.72 |

PROM=patient-reported outcome measure

| **Supplemental Table 2 (part 1/4)** | | | | | | | | | | |
| --- | --- | --- | --- | --- | --- | --- | --- | --- | --- | --- |
|  | **Barrier. N (%)** | **Overall sample** | **Time constraints** | **p-value** | **Mandated to complete** | **p-value** | **Patients dislike PROMs** | **p-value** | **Lack of integration into clinical systems** | **p-value** |
| **Variables** |  | (n=474) | (n=455) |  | (n=428) |  | (n=425) |  | (n=425) |  |
| **Sex** |  |  |  |  |  |  |  |  |  |  |
|  | **Male** | 170 (35.9%) | 162 (35.5%) |  | 168 (39.4%) |  | 152 (37.5%) |  | 164 (38.6%) |  |
|  | **Female** | 304 (64.1%) | 293 (64.5%) |  | 260 (60.6%) |  | 273 (62.6%) |  | 261 (61.4%) |  |
|  |  |  |  | 0.495 |  | 0.132 |  | **0.036** |  | 0.121 |
| **Age Group** |  |  |  |  |  |  |  |  |  |  |
|  | **20-29** | 54 (11.4%) | 54 (11.9%) |  | 54 (12.6%) |  | 54 (12.7%) |  | 54 (12.7%) |  |
|  | **30-39** | 170 (35.9%) | 168 (36.9%) |  | 162 (37.9%) |  | 167 (39.3%) |  | 152 (35.8%) |  |
|  | **40-49** | 116 (24.5%) | 109 (23.9%) |  | 104 (24.3%) |  | 100 (23.6%) |  | 103 (24.2%) |  |
|  | **50-59** | 80 (16.9%) | 73 (16.0%) |  | 59 (13.8%) |  | 59 (13.8%) |  | 75 (17.7%) |  |
|  | **60+** | 54 (11. 4%) | 51 (11.2%) |  | 49 (11.5%) |  | 45 (10.6%) |  | 41 (9.7%) |  |
|  |  |  |  | 0.074 |  | **0.023** |  | **0.019** |  | 0.528 |
| **Types of consultation** |  |  |  |  |  |  |  |  |  |  |
|  | **Public practice** | 177 (37.3%) | 170 (37.4%) |  | 167 (39.1%) |  | 176 (41.4%) |  | 164 (38.6%) |  |
|  | **Private practice** | 98 (20.7%) | 96 (21.1%) |  | 86 (20.1%) |  | 89 (20.9%) |  | 80 (18.8%) |  |
|  | **Both** | 199 (41.9%) | 189 (41.6%) |  | 175 (40.9%) |  | 160 (37.7%) |  | 181 (42.6%) |  |
|  |  |  |  | 0.505 |  | 0.344 |  | **0.009** |  | **0.028** |
| **Specialty** |  |  |  |  |  |  |  |  |  |  |
|  | **Specialist** | 392 (82.7%) | 376 (82.6%) |  | 191 (44.8%) |  | 195 (45.8%) |  | 345 (81.2%) |  |
|  |  |  |  | 0.222 |  | **0.002** |  | **0.000** |  | 0.074 |
|  | **Family Medicine** | 28 (5.9%) | 25 (5.5%) |  | 20 (4.7%) |  | 24 (5.6%) |  | 27 (6.4%) |  |
|  |  |  |  | **0.012** |  | **0.017** |  | 0.713 |  | **0.008** |
|  | **Pediatrics** | 89 (18.8%) | 89 (19.5%) |  | 58 (13.5%) |  | 60 (14.2%) |  | 80 (18.8%) |  |
|  |  |  |  | 0.710 |  | 0.396 |  | 0.338 |  | 0.874 |
|  | **Allergist** | 187 (39.5%) | 168 (36.8%) |  | 58 (13.5%) |  | 54 (12.7%) |  | 164 (38.6%) |  |
|  |  |  |  | **0.007** |  | **0.000** |  | **0.016** |  | 0.143 |
|  | **Dermatologist** | 160 (33.8%) | 147 (32.4%) |  | 148 (34.6%) |  | 115 (27.1%) |  | 132 (31.1%) |  |
|  |  |  |  | 0.630 |  | 0.500 |  | **0.016** |  | 0.056 |
| **Years as a specialist** |  |  |  |  |  |  |  |  |  |  |
|  | **1 to 9** | 191 (40.3%) | 191 (41.98%) |  | 183 (42.8%) |  | 164 (38.6%) |  | 178 (41.9%) |  |
|  | **10 to 19** | 141 (29.8%) | 132 (29.0%) |  | 103 (24.1%) |  | 121 (28.5%) |  | 124 (29.2%) |  |
|  | **20 to 29** | 74 (15.6%) | 68 (14.9%) |  | 74 (17.3%) |  | 73 (17.2%) |  | 61 (14.4%) |  |
|  | **30+** | 68 (14.3%) | 64 (14.1%) |  | 68 (15.9%) |  | 67 (15.8%) |  | 62 (14.6%) |  |
|  |  |  |  | 0.204 |  | **0.010** |  | **0.000** |  | 0.264 |

NA=not available; PROMs=patient-reported outcome measures

**Supplemental Table 2 (part 2/4)**

|  | **Barrier, n (%)** | **Overall sample** | **NA in the native language** | **p-value** | **NA for specific age groups** | **p-value** | **Sufficient understanding without PROMS** | **p-value** | **Uncertainty about reliability** | **p-value** |
| --- | --- | --- | --- | --- | --- | --- | --- | --- | --- | --- |
| **Variables** |  | (n=474) | (n=425) |  | (n=425) |  | (n=422) |  | (n=411) |  |
| **Sex** |  |  |  |  |  |  |  |  |  |  |
|  | **Male** | 170 (35.9%) | 159 (37.4%) |  | 157 (36.9%) |  | 160 (37.8%) |  | 163 (39.7%) |  |
|  | **Female** | 304 (64.1%) | 266 (62.6%) |  | 268 (63.1%) |  | 262 (62.2%) |  | 248 (60.3%) |  |
|  |  |  |  | 0.239 |  | 0.690 |  | 0.480 |  | 0.495 |
| **Age Group** |  |  |  |  |  |  |  |  |  |  |
|  | **20-29** | 54 (11.4%) | 51 (14.2%) |  | 50 (11.8%) |  | 53 (12.6%) |  | 54 (13.1%) |  |
|  | **30-39** | 170 (35.9%) | 158 (37.3%) |  | 157 (36.9%) |  | 162 (38.4%) |  | 167 (40.6%) |  |
|  | **40-49** | 116 (24.5%) | 109 (23.4%) |  | 103 (24.2%) |  | 91 (21. 6%) |  | 96 (23. 4%) |  |
|  | **50-59** | 80 (16.9%) | 61 (14.4%) |  | 72 (16.9%) |  | 65 (15.4%) |  | 59 (14.4%) |  |
|  | **60+** | 54 (11. 4%) | 46 (10.8%) |  | 43 (10.1%) |  | 51 (12.1%) |  | 35 (8.5%) |  |
|  |  |  |  | **0.037** |  | 0.104 |  | **0.040** |  | 0.039 |
| **Types of consultation** |  |  |  |  |  |  |  |  |  |  |
|  | **Public practice** | 177 (37.3%) | 148 (34.8%) |  | 148 (34.8%) |  | 162 (38.4%) |  | 167 (40.6%) |  |
|  | **Private practice** | 98 (20.7%) | 94 (22.1%) |  | 79 (18.6%) |  | 76 (18.0%) |  | 70 (17.0%) |  |
|  | **Both** | 199 (41.9%) | 183 (43.1%) |  | 198 (46.6%) |  | 184 (43.6%) |  | 174 (42.3%) |  |
|  |  |  |  | 0.239 |  | **0.029** |  | 0.471 |  | 0.348 |
| **Specialty** |  |  |  |  |  |  |  |  |  |  |
|  | **Specialist** | 392 (82.7%) | 336 (79.1%) |  | 338 (79.5%) |  | 183 (43.2%) |  | 196 (47.7%) |  |
|  |  |  |  | **0.004** |  | **0** |  | **0** |  | 0 |
|  | **Family Medicine** | 28 (5.9%) | 23 (5.4%) |  | 27 (6.4%) |  | 26 (6.2%) |  | 20 (4.9%) |  |
|  |  |  |  | 0.186 |  | 0.968 |  | 0.515 |  | 0.003 |
|  | **Pediatrics** | 89 (18.8%) | 86 (20.2%) |  | 81 (19.1%) |  | 59 (13.9%) |  | 56 (13.6%) |  |
|  |  |  |  | 0.845 |  | **0.000** |  | **0.016** |  | 0.352 |
|  | **Allergist** | 187 (39.5%) | 132 (31.1%) |  | 157 (36.9%) |  | 61 (14.4%) |  | 45 (10.9%) |  |
|  |  |  |  | **0.000** |  | **0.000** |  | **0.012** |  | 0.000 |
|  | **Dermatologist** | 160 (33.8%) | 113 (26.6%) |  | 133 (31.3%) |  | 113 (26.8%) |  | 120 (29.2%) |  |
|  |  |  |  | **0.011** |  | 0.321 |  | 0.503 |  | 0.007 |
| **Years as a specialist** |  |  |  |  |  |  |  |  |  |  |
|  | **1 to 9** | 191 (40.3%) | 183 (43.2%) |  | 182 (42.9%) |  | 176 (41.7%) |  | 163 (39.8%) |  |
|  | **10 to 19** | 141 (29.8%) | 133 (31.4%) |  | 112 (26.3%) |  | 106 (25.1%) |  | 138 (33.6%) |  |
|  | **20 to 29** | 74 (15.6%) | 59 (13.7%) |  | 68 (16.0%) |  | 73 (17.3%) |  | 70 (17.0%) |  |
|  | **30+** | 68 (14.3%) | 50 (11.8%) |  | 63 (14.8%) |  | 67 (15.9%) |  | 40 (9.7%) |  |
|  |  |  |  | **0.002** |  | **0.003** |  | 0.723 |  | 0.030 |

NA=not available; PROMs=patient-reported outcome measures

**Supplemental Table 2 (part 3/4)**

|  | **Barrier, n (%)** | **Overall sample** | **Perceived as an additional cost** | **p-value** | **Constraint doctor-patient relationship** | **p-value** | **Lack of confidence in interpreting** | **p-value** | **Feel uncomfortable** | **p-value** |
| --- | --- | --- | --- | --- | --- | --- | --- | --- | --- | --- |
| **Variables** |  | (n=474) | (n=399) |  | (n=398) |  | (n=413) |  | (n=400) |  |
| **Sex** |  |  |  |  |  |  |  |  |  |  |
|  | **Male** | 170 (35.9%) | 161 (37.6%) |  | 125 (31.4%) |  | 131 (31.7%) |  | 132 (33.0%) |  |
|  | **Female** | 304 (64.1%) | 250 (62.4%) |  | 273 (68.6%) |  | 282 (68.3%) |  | 268 (67.0%) |  |
|  |  |  |  | 0.174 |  | 0.921 |  | 0.443 |  | 0.738 |
| **Age Group** |  |  |  |  |  |  |  |  |  |  |
|  | **20-29** | 54 (11.4%) | 48 (11.7%) |  | 43 (18.4%) |  | 54 (17.9%) |  | 45 (16.3%) |  |
|  | **30-39** | 170 (35.9%) | 147 (35.8%) |  | 158 (34.7%) |  | 136 (32.9%) |  | 152 (38.0%) |  |
|  | **40-49** | 116 (24.5%) | 111 (26.9%) |  | 89 (22.4%) |  | 114 (22.8%) |  | 93 (23.3%) |  |
|  | **50-59** | 80 (16.9%) | 69 (16.8%) |  | 57 (14.3%) |  | 72 (17.4%) |  | 76 (14.0%) |  |
|  | **60+** | 54 (11.4%) | 36 (8.8%) |  | 41 (10.3%) |  | 37 (8.9%) |  | 34 (8.5%) |  |
|  |  |  |  | 0.056 |  | 0.059 |  | 0.000 |  | 0.014 |
| **Types of consultation** |  |  |  |  |  |  |  |  |  |  |
|  | **Public practice** | 177 (37.3%) | 147 (35.8%) |  | 175 (43.9%) |  | 165 (39.9%) |  | 169 (42.3%) |  |
|  | **Private practice** | 98 (20.7%) | 92 (22.4%) |  | 57 (14.3%) |  | 59 (14.3%) |  | 70 (17.5%) |  |
|  | **Both** | 199 (41.9%) | 172 (41.8%) |  | 166 (41.7%) |  | 189 (45.8%) |  | 161 (40.3%) |  |
|  |  |  |  | 0.004 |  | 0.846 |  | 0.025 |  | 0.300 |
| **Specialty** |  |  |  |  |  |  |  |  |  |  |
|  | **Specialist** | 392 (82.7%) | 292 (73.2%) |  | 260 (65.3%) |  | 286 (69.3%) |  | 287 (71.8%) |  |
|  |  |  |  | 0.000 |  | 0.000 |  | 0.000 |  | 0.000 |
|  | **Family Medicine** | 28 (5.9%) | 22 (5.5%) |  | 28 (7.0%) |  | 28 (6.8%) |  | 21 (5.3%) |  |
|  |  |  |  | 0.001 |  | 0.018 |  | 0.105 |  | 0.035 |
|  | **Pediatrics** | 89 (18.8%) | 37 (9.3%) |  | 45 (11.3%) |  | 67 (16.2%) |  | 70 (17.5%) |  |
|  |  |  |  | 0.378 |  | 0.134 |  | 0.499 |  | 0.217 |
|  | **Allergist** | 187 (39.5%) | 58 (14.5%) |  | 65 (16.3%) |  | 109 (26.4%) |  | 118 (29.5%) |  |
|  |  |  |  | 0.000 |  | 0.000 |  | 0.000 |  | 0.000 |
|  | **Dermatologist** | 160 (33.8%) | 39 (9.8%) |  | 97 (24.4%) |  | 106 (25.7%) |  | 101 (25.3%) |  |
|  |  |  |  | 0.240 |  | 0.052 |  | 0.002 |  | 0.033 |
|  |  |  |  |  |  |  |  |  |  |  |
| **Years as a specialist** |  |  |  |  |  |  |  |  |  |  |
|  | **1 to 9** | 191 (40.3%) | 154 (37.4%) |  | 171 (42.9%) |  | 190 (46.0%) |  | 186 (46.5%) |  |
|  | **10 to 19** | 141 (29.8%) | 117 (28.4%) |  | 118 (29.7%) |  | 104 (25.2%) |  | 107 (26.8%) |  |
|  | **20 to 29** | 74 (15.6%) | 72 (17.5%) |  | 69 (17.3%) |  | 74 (17.9%) |  | 68 (17.0%) |  |
|  | **30+** | 68 (14.3%) | 68 (16.6%) |  | 40 (10.1%) |  | 45 (10.9%) |  | 39 (9.8%) |  |
|  |  |  |  | 0.003 |  | 0.002 |  | 0.000 |  | 0.002 |

NA=not available; PROMs=patient-reported outcome measures

**Supplemental Table 2 (part 4/4).**

|  | **Barrier**  **N (%)** | **Overall sample** | **NA for obtaining the information** | **p-value** | **Too complicated to fill in** | **p-value** | **Too complicated to evaluate/score** | **p-value** |
| --- | --- | --- | --- | --- | --- | --- | --- | --- |
| **Variables** |  | (n=474) | (n=409) |  | (n=406) |  | (n=403) |  |
| **Sex** |  |  |  |  |  |  |  |  |
|  | **Male** | 170 (35.9%) | 158 (38.6%) |  | 150 (36.9%) |  | 126 (31.3%) |  |
|  | **Female** | 304 (64.1%) | 251 (61.4%) |  | 256 (63.1%) |  | 277 (68.7%) |  |
|  |  |  |  | 0.196 |  | 0.649 |  | 0.074 |
| **Age Group** |  |  |  |  |  |  |  |  |
|  | **20-29** | 54 (11.4%) | 46 (18.6%) |  | 51 (12.5%) |  | 52 (15.5%) |  |
|  | **30-39** | 170 (35.9%) | 151 (32.0%) |  | 160 (39.3%) |  | 165 (40.8%) |  |
|  | **40-49** | 116 (24.5%) | 91 (22.3%) |  | 88 (21.9%) |  | 102 (22.8%) |  |
|  | **50-59** | 80 (16.9%) | 67 (16.4%) |  | 64 (15.7%) |  | 58 (14.4%) |  |
|  | **60+** | 54 (11.4%) | 44 (10.8%) |  | 43 (10.6%) |  | 26 (6.4%) |  |
|  |  |  |  | 0.050 |  | 0.091 |  | 0.007 |
| **Types of consultation** |  |  |  |  |  |  |  |  |
|  | **Public practice** | 177 (37.3%) | 143 (34.9%) |  | 153 (37.7%) |  | 176 (43.7%) |  |
|  | **Private practice** | 98 (20.7%) | 67 (16.4%) |  | 92 (22.7%) |  | 81 (20.1%) |  |
|  | **Both** | 199 (41.9%) | 199 (48.7%) |  | 161 (39.7%) |  | 146 (36.2%) |  |
|  |  |  |  | 0.000 |  | 0.584 |  | 0.884 |
| **Specialty** |  |  |  |  |  |  |  |  |
|  | **Specialist** | 392 (82.7%) | 304 (74.3%) |  | 302 (74.4%) |  | 298 (73.9%) |  |
|  |  |  |  | 0.003 |  | 0.082 |  | 0.240 |
|  | **Family Medicine** | 28 (5.9%) | 26 (6.4%) |  | 26 (6.4%) |  | 26 (6.5%) |  |
|  |  |  |  | 0.020 |  | 0.000 |  | 0.020 |
|  | **Pediatrics** | 89 (18.8%) | 67 (16.4%) |  | 79 (19.5%) |  | 71 (17.6%) |  |
|  |  |  |  | 0.851 |  | 0.932 |  | 0.395 |
|  | **Allergist** | 187 (39.5%) | 114 (27.9%) |  | 127 (31.3%) |  | 123 (30.5%) |  |
|  |  |  |  | 0.000 |  | 0.001 |  | 0.015 |
|  | **Dermatologist** | 160 (33.8%) | 120 (29.3%) |  | 117 (28.8%) |  | 120 (29.8%) |  |
|  |  |  |  | 0.235 |  | 0.413 |  | 0.695 |
|  |  |  |  |  |  |  |  |  |
| **Years as a specialist** |  |  |  |  |  |  |  |  |
|  | **1 to 9** | 191 (40.3%) | 184 (44.9%) |  | 170 (41.9%) |  | 191 (47.4%) |  |
|  | **10 to 19** | 141 (29.8%) | 117 (28.6%) |  | 122 (30.1%) |  | 115 (28.5%) |  |
|  | **20 to 29** | 74 (15.6%) | 58 (14.2%) |  | 58 (14.3%) |  | 58 (14.4%) |  |
|  | **30+** | 68 (14.3%) | 50 (12.2%) |  | 56 (13.8%) |  | 39 (9.7%) |  |
|  |  |  |  | 0.003 |  | 0.732 |  | 0.001 |

NA=not available; PROMs=patient-reported outcome measures

**Supplemental Table 3 (part 1/3). Results of logistic regression: correlates of barriers to PROMs use in CU and AD patients.**

|  | **Barriers Odds Ratio (SE)** | | | | |
| --- | --- | --- | --- | --- | --- |
| **VARIABLES** | **Time constraints** | **Not mandated to complete** | **Sufficient understanding without PROMS** | **Patients dislike PROMs** | **Uncertainty about reliability** |
| **Sex (Ref = Male)** |  |  |  |  |  |
| **Female** | 0.941 | 0.627** | 0.797 | 0.809 | 0.670* |
|  | (0.241) | (0.132) | (0.164) | (0.168) | (0.143) |
| **Age Group (Ref=20-29)** |  |  |  |  |  |
| **30-39** | 0.393* | 0.531* | 0.963 | 1.543 | 0.694 |
|  | (0.213) | (0.201) | (0.342) | (0.576) | (0.247) |
| **40-49** | 0.330* | 0.454* | 0.924 | 1.429 | 1.151 |
|  | (0.206) | (0.206) | (0.404) | (0.639) | (0.511) |
| **50-59** | 0.209** | 0.448 | 1.109 | 1.030 | 0.947 |
|  | (0.152) | (0.250) | (0.605) | (0.562) | (0.530) |
| **60+** | 0.0762*** | 0.660 | 1.493 | 1.185 | 0.859 |
|  | (0.0693) | (0.466) | (1.041) | (0.823) | (0.623) |
| **Type of consultation (Re=Public practice)** | |  |  |  |  |
| **Private practice** | 0.907 | 0.841 | 0.721 | 0.714 | 0.640 |
|  | (0.314) | (0.232) | (0.199) | (0.198) | (0.185) |
| **Both public and private** | 0.878 | 0.919 | 1.060 | 0.516*** | 1.064 |
|  | (0.246) | (0.210) | (0.239) | (0.118) | (0.247) |
| **Specialist (Ref=No specialist)** | 3.244** | 0.875 | 0.384*** | 0.554 | 0.805 |
|  | (1.549) | (0.332) | (0.142) | (0.216) | (0.299) |
| **Specialty (Ref=Physicians outside of the specialties below)** | | |  |  |  |
| **Family medicine** | 5.337 | 1.928 | 2.583** | 1.805 | 1.648 |
|  | (5.553) | (0.863) | (1.113) | (0.824) | (0.718) |
| **Pediatrics** | 1.259 | 1.251 | 2.151*** | 0.880 | 0.715 |
|  | (0.449) | (0.343) | (0.594) | (0.240) | (0.209) |
| **Allergist** | 0.425*** | 0.448*** | 0.633* | 0.864 | 0.519** |
|  | (0.140) | (0.114) | (0.160) | (0.217) | (0.139) |
| **Dermatologist** | 0.465** | 0.625* | 1.072 | 0.767 | 0.473*** |
|  | (0.156) | (0.166) | (0.285) | (0.202) | (0.133) |
| **Years being a specialist (Ref=9 or less)** | |  |  |  |  |
| **10-19** | 0.735 | 1.025 | 1.102 | 0.708 | 0.658 |
|  | (0.260) | (0.293) | (0.314) | (0.203) | (0.196) |
| **20-29** | 1.101 | 0.909 | 0.871 | 0.861 | 0.603 |
|  | (0.589) | (0.395) | (0.383) | (0.371) | (0.273) |
| **30+** | 3.059 | 0.950 | 0.967 | 0.858 | 0.632 |
|  | (2.281) | (0.557) | (0.571) | (0.498) | (0.391) |
| **Constant** | 9.161*** | 5.494*** | 2.126** | 3.745*** | 2.538*** |
|  | (4.836) | (2.161) | (0.747) | (1.394) | (0.907) |
| **Observations** | 468 | 468 | 468 | 468 | 468 |

AD=atopic dermatitis; CU=chronic urticaria; PROM=patient-reported outcome measure; Ref=reference; * p<0.1; **p<0.05; *** p<0.01. The values enclosed in parentheses are the standard error values of the regression models.

**Supplemental Table 3 (part 2/3). Logistic regression results: correlates of barriers to PROM use in CU and AD patients.**

|  | **Barriers Odd Ratio (SE) (n=371)** | | | | |
| --- | --- | --- | --- | --- | --- |
| **VARIABLES** | **Perceived as an additional cost** | **Constraint doctor-patient relationship** | **Lack of integration into clinical systems** | **Lack of confidence in interpreting** | **Feel uncomfortable** |
| **Sex (Ref = Male)** |  | | | | |
| **Female** | 0.880 | 1.338 | 0.716 | 1.409 | 1.179 |
|  | (0.211) | (0.360) | (0.148) | (0.320) | (0.268) |
| **Age Group (Ref=20-29)** |  |  |  |  |  |
| **30-39** | 1.215 | 0.683 | 0.704 | 0.518* | 0.832 |
|  | (0.473) | (0.271) | (0.252) | (0.190) | (0.302) |
| **40-49** | 1.894 | 0.740 | 0.810 | 0.860 | 0.812 |
|  | (0.959) | (0.394) | (0.354) | (0.395) | (0.373) |
| **50-59** | 3.459* | 0.733 | 0.828 | 0.998 | 0.577 |
|  | (2.257) | (0.515) | (0.448) | (0.571) | (0.340) |
| **60+** | 3.516 | 1.430 | 0.254* | 0.938 | 0.686 |
|  | (2.921) | (1.334) | (0.184) | (0.701) | (0.535) |
| **Type of consultation (Ref=Public practice)** |  |  |  |  |  |
| **Private practice** | 0.583 | 0.568 | 0.719 | 0.563* | 0.710 |
|  | (0.202) | (0.212) | (0.194) | (0.176) | (0.215) |
| **Both public and private** | 1.219 | 0.915 | 0.983 | 1.227 | 0.878 |
|  | (0.318) | (0.260) | (0.221) | (0.297) | (0.214) |
| **Specialist (Ref= No specialist)** | 0.428** | 0.660 | 0.970 | 0.425* | 0.564 |
|  | (0.174) | (0.284) | (0.357) | (0.163) | (0.215) |
| **Specialty (Ref=Physicians outside of specialties below)** |  | | | | |
| **Family medicine** | 2.177 | 1.023 | 2.048 | 1.627 | 1.864 |
|  | (1.035) | (0.544) | (0.930) | (0.714) | (0.800) |
| **Pediatrics** | 0.448** | 0.496 | 1.059 | 0.966 | 1.103 |
|  | (0.176) | (0.214) | (0.290) | (0.296) | (0.338) |
| **Allergist** | 0.441** | 0.194*** | 0.905 | 0.425*** | 0.623* |
|  | (0.141) | (0.0716) | (0.227) | (0.120) | (0.175) |
| **Dermatologist** | 0.723 | 0.393** | 0.752 | 0.545** | 0.658 |
|  | (0.232) | (0.145) | (0.198) | (0.162) | (0.197) |
| **Years being a specialist (Ref=9 or less)** |  |  |  |  |  |
| **10-19** | 0.845 | 1.692 | 0.900 | 0.857 | 1.024 |
|  | (0.291) | (0.651) | (0.253) | (0.270) | (0.320) |
| **20-29** | 0.422 | 2.625 | 0.714 | 1.280 | 1.743 |
|  | (0.236) | (1.573) | (0.307) | (0.593) | (0.831) |
| **30+** | 0.569 | 1.093 | 2.086 | 0.730 | 1.112 |
|  | (0.406) | (0.949) | (1.297) | (0.469) | (0.762) |
| **Constant** | 0.820 | 0.737 | 2.956*** | 1.938* | 0.977 |
|  | (0.303) | (0.281) | (1.064) | (0.710) | (0.345) |
| **Observations** | 468 | 468 | 468 | 468 | 468 |

AD=atopic dermatitis; CU=chronic urticaria; PROM=patient-reported outcome measure; Ref=reference; * p<0.1; **p<0.05; *** p<0.01. The values enclosed in parentheses are the standard error values of the regression models.

**Supplemental Table 3 (part 3/3). Logistic regression: correlates of barriers to PROMs use in CU and AD patients.**

|  | **Barriers Odds Ratio (SE) (n=371)** | | | | |
| --- | --- | --- | --- | --- | --- |
| **VARIABLES** | **Not available in the native language** | **Not available for specific age groups** | **Not suitable for obtaining the information** | **Too complicated to fill in** | **Too complicated to evaluate/score** |
| **Sex (Ref=Male)** |  |  |  |  |  |
| **Female** | 0.879 | 0.854 | 0.810 | 0.856 | 1.189 |
|  | (0.184) | (0.175) | (0.182) | (0.183) | (0.261) |
| **Age Group (Ref=20-29)** |  |  |  |  |  |
| **30-39** | 0.648 | 1.195 | 0.416** | 1.411 | 1.054 |
|  | (0.227) | (0.421) | (0.151) | (0.522) | (0.373) |
| **40-49** | 0.667 | 1.351 | 0.592 | 1.030 | 0.786 |
|  | (0.290) | (0.586) | (0.274) | (0.475) | (0.350) |
| **50-59** | 0.776 | 0.871 | 0.931 | 1.154 | 0.671 |
|  | (0.427) | (0.475) | (0.544) | (0.663) | (0.384) |
| **60+** | 1.327 | 0.832 | 1.132 | 0.901 | 0.324 |
|  | (0.970) | (0.582) | (0.871) | (0.663) | (0.249) |
| **Type of consultation (Ref=Public practice)** |  |  |  |  |  |
| **Private practice** | 1.154 | 0.954 | 0.846 | 1.202 | 0.826 |
|  | (0.318) | (0.260) | (0.268) | (0.340) | (0.235) |
| **Both public and private** | 1.233 | 1.730** | 1.663** | 1.003 | 0.680 |
|  | (0.283) | (0.393) | (0.414) | (0.238) | (0.162) |
| **Specialist (Ref=No specialist)** | 1.420 | 0.406** | 0.819 | 0.412** | 0.663 |
|  | (0.523) | (0.151) | (0.318) | (0.156) | (0.249) |
| **Specialty (Ref=Physicians outside of the specialties below)** |  |  |  |  |  |
| **Family medicine** | 1.541 | 1.907 | 1.265 | 1.638 | 1.058 |
|  | (0.671) | (0.834) | (0.591) | (0.697) | (0.465) |
| **Pediatrics** | 1.154 | 2.556*** | 0.892 | 1.329 | 1.107 |
|  | (0.321) | (0.722) | (0.282) | (0.384) | (0.330) |
| **Allergist** | 0.405*** | 0.944 | 0.470*** | 0.631* | 0.639* |
|  | (0.107) | (0.240) | (0.135) | (0.168) | (0.174) |
| **Dermatologist** | 0.401*** | 1.212 | 0.593* | 0.896 | 0.808 |
|  | (0.113) | (0.322) | (0.180) | (0.252) | (0.231) |
| **Years being a specialist (Ref=9 or less)** |  |  |  |  |  |
| **10-19** | 1.118 | 0.713 | 0.927 | 1.371 | 1.064 |
|  | (0.320) | (0.202) | (0.297) | (0.410) | (0.315) |
| **20-29** | 0.726 | 1.598 | 0.665 | 1.453 | 1.446 |
|  | (0.325) | (0.704) | (0.327) | (0.675) | (0.673) |
| **30+** | 0.467 | 0.972 | 0.443 | 1.752 | 1.697 |
|  | (0.295) | (0.576) | (0.297) | (1.093) | (1.098) |
| **Constant** | 1.433 | 1.459 | 1.370 | 0.864 | 0.971 |
|  | (0.495) | (0.504) | (0.485) | (0.305) | (0.338) |
| **Observations** | 468 | 468 | 468 | 468 | 468 |

AD=atopic dermatitis; CU=chronic urticaria; PROM=patient-reported outcome measure; Ref=reference; * p<0.1; **p<0.05; *** p<0.01. The values enclosed in parentheses are the standard error values of the regression models.
